# GNAS promotes inflammation-related hepatocellular carcinoma progression by promoting STAT3 activation

**DOI:** 10.1186/s11658-020-00204-1

**Published:** 2020-02-24

**Authors:** Hongda Ding, Xixia Zhang, Yang Su, Changjun Jia, Chaoliu Dai

**Affiliations:** 1 Department of General Surgery, Shengjing Hospital of China Medical University, No. 36 Sanhao Road, Shenyang, 110004 China; 2 Department of Otolaryngology Head and Neck Surgery, Shengjing Hospital of China Medical University, No. 36 Sanhao Road, Shenyang, 110004 China

**Keywords:** Hepatocellular carcinoma, Lipopolysaccharides, G-protein alpha-subunit, Signal transducer and activator of transcription 3, Interleukin-6

## Abstract

**Background:**

Hepatocellular carcinoma (HCC) is still the most common cause of cancer-related mortality worldwide and accumulating studies report that HCC is frequently linked to chronic inflammation. G-protein alpha-subunit (GNAS)-activating mutations have recently been reported to form a rare subgroup of inflammatory liver tumors. In this study, we investigated the roles of GNAS in inflammation-related HCC progression and its underlying mechanism.

**Methods:**

Lipopolysaccharides (LPS) and diethylnitrosamine were employed to stimulate HCC cells to an induced inflammatory response. qRT-PCR, immunohistochemistry and immunoblotting were performed to detect the expression of GNAS in HCC tissues and cell lines. Expression levels of proinflammatory cytokines were detected by qRT-PCR and ELISA. N6-methyladenosine (m6A) methylation of GNAS mRNA was detected by RNA-binding protein immunoprecipitation (RIP). Transcription factors activation profiling plate array was performed to investigate the underlying mechanism in GNAS promoting interleukin-6 (IL-6) expression in HCC cells. HCC cell invasion was determined by transwell assay in vitro, and tumorigenesis was assessed with a subcutaneous xenograft mouse model of HCC.

**Results:**

We found that LPS stimulation promotes GNAS expression in HCC cells through increasing m6A methylation of GNAS mRNA. The high expression level of GNAS promotes LPS-induced HCC cell growth and invasion by interacting with signal transducer and activator of transcription 3 (STAT3). Furthermore, GNAS knockdown inhibits LPS induced-IL-6 expression in HCC cells by suppressing STAT3 activation. Moreover, we found that GNAS promotes LPS-induced STAT3 activation in HCC cells through inhibiting long non-coding RNA TPTEP1 interacting with STAT3. In addition, GNAS expression promotes HCC development in mice and is related to poor survival.

**Conclusions:**

Our findings for the first time indicate a tumor-promoting role of GNAS in inflammation-related HCC progression and provide a novel potential target for HCC therapy.

## Background

Hepatocellular carcinoma (HCC) is a highly aggressive malignancy and the most common form of liver cancer, causing over 780,000 deaths worldwide each year [1–3]. Despite great advances in HCC therapy, the treatment effect for HCC patients is still not satisfactory, with low 5-year survival and a high recurrence rate [4, 5]. Nowadays, numerous studies report that HCC is frequently linked to chronic inflammation [6–8]. Therefore, clarifying the molecular mechanism of inflammation in HCC progression and searching for newly therapeutic targets for HCC are highly urgent.

Tumor-promoting inflammation and avoidance of the immune system have been reported to be among the new hallmarks of cancer [9, 10]. Inflammation in the tumor microenvironment not only promotes tumorous cell proliferation and metastasis, but also triggers chemotherapy tolerance [11–13]. Cytokines, such as tumor necrosis factor alpha (TNF-α), interleukin-6 (IL-6), and transforming growth factor-beta (TGF-β), are the major mediators which are responsible for interchanging among cells in the tumor microenvironment [14–16]. Particularly, IL-6 has been reported to be one of the most important pro-tumor factors in HCC progression [17]. For example, mice with IL-6 gene knockout develop much less HCC in response to diethylnitrosamine (DEN) [18]. Now, although exploring the pathological mechanisms of tumor-related inflammatory responses attracts much attention, the molecular mechanisms in inflammation-related HCC progression are still not completely known.

The GNAS gene encodes the alpha-subunit of the stimulatory G protein (Gsα), which functions to regulate neurotransmitters and many hormones through generating cAMP [19, 20]. GNAS mutations are reported to be highly associated with McCune-Albright syndrome [21, 22]. Recently, GNAS-activating mutations have been reported to constitute a rare subgroup of inflammatory liver cancer with signal transducer and activator of transcription 3 (STAT3) activation [23]. However, whether GNAS is involved in inflammation-related HCC progression and its underlying mechanism remain unclear.

In this study, we investigated the roles of GNAS in inflammation-related HCC progression and its underlying mechanism. This study revealed that LPS stimulation promotes GNAS expression in HCC cells through increasing N6-methyladenosine (m6A) methylation of GNAS mRNA. The high expression level of GNAS promotes LPS-induced HCC cell growth and invasion by interacting with STAT3. Furthermore, GNAS knockdown inhibits LPS induced-IL-6 expression in HCC cells by suppressing STAT3 activation. Moreover, we found that GNAS promotes LPS-induced STAT3 activation in HCC cells through inhibiting long non-coding RNA TPTEP1 interacting with STAT3. Our findings for the first time suggest a tumor-promoting role of GNAS in inflammation-related HCC progression and afford a novel potential target for HCC therapy.

## Methods

### Ethics statement

This study was approved by the Ethics Committee of Shengjing Hospital of China Medical University. All study participants provided written informed consent.

### Collection of specimens

A total of 12 matched samples of primary HCC and adjacent non-cancerous liver tissues were obtained from Shengjing Hospital of China Medical University. This study was approved by the ethics committee of our hospital, and all participants signed informed consent forms in this study. No patients had received chemotherapy or radiotherapy prior to surgery. HCC and normal tissue specimens were obtained immediately after surgical resection and stored at − 80 °C for further analysis.

### Cells, siRNAs and reagents

The human HCC cells, including HepG2, QGY-7703, Huh-7, and MHCC97h, and HL-7702 human normal liver cells were stored in our laboratory, and were cultured as described in our previously published study [24, 25]. The sequences of siRNAs against GNAS (si-GNAS), si-YTHDF1, and scrambled siRNA (NC) are listed in Table 1. siRNAs were synthesized by Shanghai GenePharma Co., Ltd. GNAS was amplified by PCR, and then subcloned into pCMV-Myc vector. The primers for GNAS amplification are listed in Table 1. pCMV-Flag-STAT3 vector was stored in our laboratory [25]. Lipopolysaccharides (LPS), and the specific NF-κB inhibitor ammonium pyrrolidine dithiocarbamate (PDTC) were purchased from Beyotime (Shanghai, China). The specific STAT3 inhibitor C188–9 was purchased from Selleck. N-Nitrosodiethylamine (DEN) was purchased from Meilunbio (Dalian, China).

**Table 1:** Primers used in this study (F: forward primer; R: reverse primer)

Name	Sequence	
Primers for GNAS constructs		
GNAS F	5′-GCCATGGAGGCCCGAATTCGGGTTCGTTGCAACAAATTGAT-3′	pCMV-Myc-GNAS
GNAS R	5′-GGCCGCGGTACCTCGAGGTTCGTTGCAACAAATT-3′	
Primers for qRT-PCR		
GNAS F	5′-TGCCTCGGGAACAGTAAGAC-3′	
GNAS R	5′-GCCGCCCTCTCCATTAAAC-3′	
IL-6 F	5′-ACTCACCTCTTCAGAACGAATTG-3′	
IL-6 R	5′-CCATCTTTGGAAGGTTCAGGTTG-3′	
TNFα F	5′-CCTCTCTCTAATCAGCCCTCTG-3′	
TNFα R	5′-GAGGACCTGGGAGTAGATGAG-3′	
IL-1β F	5′-ATGATGGCTTATTACAGTGGCAA-3′	
IL-1β R	5′-GTCGGAGATTCGTAGCTGGA-3′	
IL-8 F	5′-TTTTGCCAAGGAGTGCTAAAGA-3′	
IL-8 R	5′-AACCCTCTGCACCCAGTTTTC-3′	
IL-10 F	5′-CCTCCGTCTGTGTGGTTTGAA-3′	
IL-10 R	5′-CACTGCGGTAAGGTCATAGGA-3′	
Bcl-xl F	5′-GAGCTGGTGGTTGACTTTCTC-3′	
Bcl-xl R	5′-TCCATCTCCGATTCAGTCCCT-3′	
Cyclin D F	5′-GCTGCGAAGTGGAAACCATC-3′	
Cyclin D R	5′-CCTCCTTCTGCACACATTTGAA-3′	
Mcl1 F	5′-TGCTTCGGAAACTGGACATCA-3′	
Mcl1 R	5′-TAGCCACAAAGGCACCAAAAG-3′	
GAPDH F	5′-TCAACAGCAACTCCCACTCTTCCA-3′	
GAPDH R	5′-ACCCTGTTGCTGTAGCCGTATTCA-3′	
Sequences of siRNAs		
si-GNAS	5′-TCGAAGATTGAGGACTACTTTCC-3′	
si-YTHDF1	5′-CCUACGGACAGCUCAGUAAT −3′	
scrambled siRNA	5′-UUCUCCGAACGUGUCACGUTT-3′	
Primers used for ChIP		
IL-6 promoter F	5′-GCTGGCTAGCCTGCTTATGTCAG-3′	
IL-6 promoter R	5′-TCATTGAGGCTAGCGCTAAGAA-3′	

### Quantitative real-time PCR (qRT-PCR)

Total RNA of HCC cells was extracted, reverse-transcribed into cDNA, and then used to perform qRT-PCR as described in our previously published study [24, 25]. qRT-PCR primers for GNAS, IL-6, TNFα, IL-1β, IL-8, IL-10, Bcl-xl, cyclin D, Mcl1, and GAPDH are listed in Table 1. The obtained data were normalized to GAPDH expression levels in each sample.

### Enzyme-linked immunosorbent assay (ELISA)

HepG2 cells were transfected with specific siRNA for 24 h and then treated with 5 μg/ml LPS for 12 h. The culture supernatants were collected and IL-6 protein expression levels were measured using an ELISA kit (Abcam, ab178013), according to the manufacturer’s instructions.

### Subcellular fractionation and Western blotting

The cytoplasm and nuclear fraction of cells were extracted using a nuclear and cytoplasmic protein extraction kit (Beyotime, Shanghai, China), according to the manufacturer’s instructions. Whole cell lysates or the nuclear/cytoplasm fractions were subjected to SDS-PAGE and immunoblotting, as described in our previously published study [24, 25]. Primary antibodies against STAT3 (Abcam, ab119352), phosphorylated STAT3 (p-STAT3) (Abcam, ab76315), GAPDH (Abcam, ab181602), GNAS (Proteintech, 10,150–2-AP), YTHDF1 (Abcam, ab220162), YTHDF2 (Abcam, ab220163), YTHDF3 (Abcam, ab220161), P65 (Proteintech, 10,745–1-AP), phosphorylated P65 (pp65) (Abcam, ab76302), JAK1 (Abcam, ab133666), and JAK2 (Abcam, ab108596) were used.

### RNA-binding protein immunoprecipitation (RIP) assay

RIP assays were performed essentially as described in our previously published study [24, 25]. In brief, cells were lysed using polysome lysis buffer (5 mM HEPES (pH 7.4), 85 mM KCl, 1 mM DTT, 5 mM PMSF, 0.5% NP40, supplemented with RNase inhibitors (Invitrogen, USA) and PIC (protease inhibitors cocktail, Roche, Switzerland)) on ice for 10 min. After centrifugation, the supernatant was collected with 10% of the lysate serving as “input”. The remainder of the lysate was incubated with 50 μl of protein A/G magnetic beads (Life Technologies, USA) coupled with 2 μg of primary antibodies rotated overnight at 4 °C with IgG antibody as the control. RNA was isolated using TRIzol (Invitrogen, USA) and reverse-transcribed into cDNA for qRT-PCR detection using a Takara SYBR green kit (Takara, Japan). Primary antibodies against YTHDF1 (Abcam, ab220162), YTHDF2 (Abcam, ab220163), YTHDF3 (Abcam, ab220161), and N6-methyladenosine (m6A) (Abcam, ab220161) were used.

### Chromatin immunoprecipitation (ChIP)

ChIPs were performed using an EZ-Magna ChIP Chromatin Immunoprecipitation Kit (Millipore, USA), as described in our previously published study [24, 25]. Primary antibodies against STAT3 (Abcam, USA) were used. Purified DNA was analyzed by qPCR. The primers are listed in Table 1.

### Polysome fractionation

Polysome fractionations were performed as described previously [26]. Briefly, HepG2 cells (one 10 cm culture dish) were treated with 100 mg/ml cycloheximide (Cayman) for 10 min at 37 °C. Then, cells were harvested and 200 μl of cytoplasmic extract was layered onto 10–50% sucrose gradient and centrifuged at 39,000 rpm in a Beckman SW-41Ti rotor for 3 h at 4 °C. Samples were collected from the top of the gradient into 15 fractions. Collected fractions were then analyzed by qPCR.

### Generation of knockout cell line with CRISPR/Cas9

Guide RNA sequences for CRISPR/Cas9 were designed at the CRISPR design web site (http://crispr.mit.edu/). Insert oligonucleotides for human GNAS gRNA are CGGUUGAAAAAACAUGUUUCAA. The complementary oligonucleotides for guide RNAs (gRNAs) were annealed, and cloned into pX459 CRISPR/Cas9-Puro vector (Addgene, Cambridge, MA). HepG2 cells were transfected with pX459/gRNA with Lip3000, according to the manufacturer’s instructions. Two days after transfection, cells were treated with 1 μg/ml of puromycin for 3 days. After 2 weeks, colonies were isolated with the cloning cylinders, and the GNAS sequences were analyzed with T7 endonuclease (T7E1) assay, DNA sequencing and Western blot.

### TF activation profiling plate Array

The nucleoprotein extracts of HepG2 cells were prepared and subjected to TF Activation Profiling Plate Array (Signosis, Inc., Santa Clara, CA, USA), according to the manufacturer’s protocol. The TF Activation Profiling Plate Array was used to determine the activities of 96 TFs in one plate. The activity of each TF was automatically recorded and 1.5 was set as the threshold value for screening over-activated TFs.

### Matrigel invasion assay

Matrigel invasion assay was performed as described in our previously published study [24, 25].

### Cell proliferation assay

Cell proliferation was detected by the MTT assay kit (Beyotime, Shanghai, China), as described in our previously published study [24, 25].

### RNA pull-down assay

RNA pull-down assays were performed essentially as described in our previously published study [24, 25].

### Co-immunoprecipitation (co-IP) assay and mass spectrometry

Co-IP was performed as previously described [27]. Briefly, the cells were lysed and centrifuged for the supernatant. One tenth of the supernatant was retained for the immunoblot of input, and the rest was incubated with anti-STAT3 (Abcam, ab119352), anti-GNAS (Proteintech, 10,150–2-AP), anti-Flag (Abcam, ab205606), anti-Myc (Abcam, ab32), or rabbit/mouse IgG at 4 °C overnight, followed by further incubation with 10 μl of protein A-agarose beads (Cell Signaling Technology) for another 4 h. The bound proteins were subjected to washing three times for 30 min each and then eluted by boiling for 5 min in the loading buffer. Immunocomplexes were analyzed by SDS-PAGE electrophoresis and Western blotting, and the gel was then stained with the Fast Silver Stain Kit (Beyotime, Shanghai, China). Proteins specially interacting with STAT3 were identified by reverse-phase liquid chromatography coupled with tandem mass spectrometry (ACQUITY UPLC UPLC-QTOF).

### Tumor formation in nude mice

Twelve 4-week-old male BALB/c nude mice were divided into 2 groups randomly. Each group was composed of 6 mice that were injected with 2 × 10^6^ HepG2 cells (WT), or GNAS knockout HepG2 cells (GNAS-cas9). Three weeks later, all mice were killed and the weight of each tumor was measured. Tumor tissues were integrally stripped out. All animal studies were approved by the Animal Ethics Committee of China Medical University and experiments were conducted according to the National Institutes of Health Guide for the Care and Use of Laboratory Animals.

### Immunohistochemistry

Paraffin-embedded sections of xenograft tumors from the nude mice were dewaxed with 100, 90, 70, and 50% alcohol solutions (5 min each at 37 °C), followed by heat-induced repair in 0.01 mol/l citrate buffer (pH 6.0), 20 min of endogenous peroxidase inhibition with 0.3% hydrogen peroxide, 30 min of incubation at room temperature in 20% normal goat serum and overnight incubation at 4 °C with anti-pSTAT3 antibody or anti-GNAS antibody. The sections were then incubated for an additional 1 h at 37 °C, washed with 0.01 mol/l PBS and incubated for 20 min at 37 °C with HRP-conjugated secondary antibody. After development with 3,3′-diaminobenzidine reagent for 5 min at room temperature, sections were observed for staining under a light microscope. Finally, hematoxylin was used for 30 s of counterstaining; sections were then rinsed with running water for 5 min, hyalinized and mounted with neutral resin prior to observation under a light microscope.

### Statistical analysis

Data were statistically analyzed and graphed using GraphPad Prism 5 (GraphPad Software, San Diego, CA, USA). All results were presented as mean values ± standard deviations. Statistically significant differences between groups were determined by Student’s t-test. *P < 0.05.

## Results

### LPS stimulation promotes GNAS expression in HCC cells, and GNAS knockdown inhibits LPS-induced IL-6 expression

**Fig. 1:**
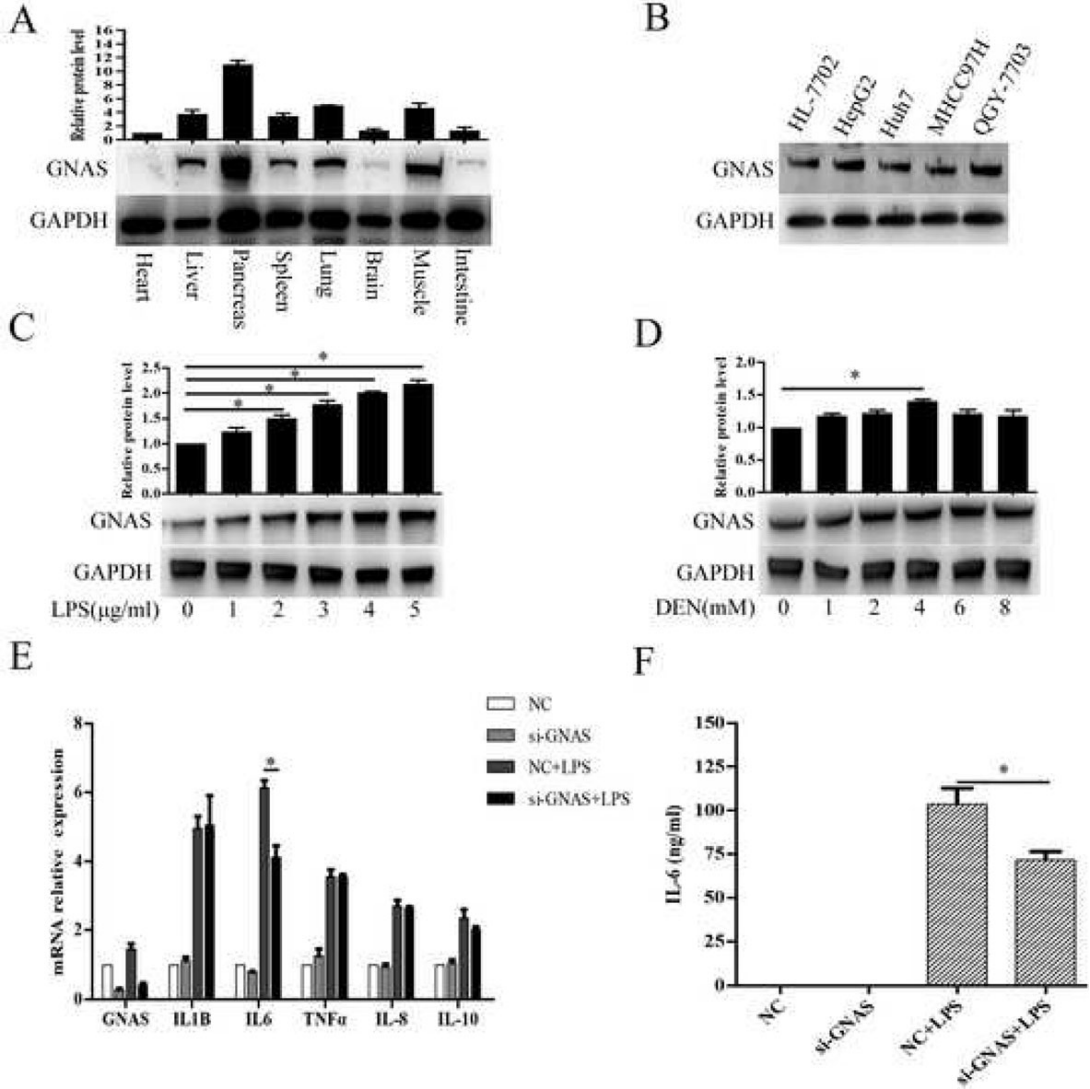
LPS stimulation promotes GNAS expression in HCC cells, and GNAS knockdown inhibits LPS-induced IL-6 expression. **a** GNAS protein expression levels in different tissues were detected by Western blotting. **b** GNAS protein expression levels in the indicated HCC cell lines and HL-7702 human normal liver cells were detected by Western blotting. **c** and **d** HepG2 cells were treated with the indicated LPS (**c**) or DEN (**d**) for 12 h, and then GNAS protein expression levels were detected by Western blotting. **e** and **f** HepG2 cells were transfected with si-NC or si-GNAS for 24 h and then treated with LPS (5 μg/ml) for 12 h. The relative expression levels of the indicated mRNAs were analyzed by qRT-PCR (**e**). The expression levels of IL-6 in the culture supernatants were measured by ELISA (**f**). Data are represented as means ± SD (*n* = 3; ^*^represents *P* < 0.05)

HCC is frequently linked to chronic inflammation [6–8], and GNAS-activating mutations have been reported to form a rare subgroup of inflammatory liver tumors [23]. In the present study, we investigated the roles of GNAS in inflammation-related HCC progression and its related mechanism. We first detected the protein expression levels of GNAS in various organs of mouse in vivo*.* Western blotting analysis showed that GNAS is highly expressed in liver, pancreas, spleen, lung and intestine tissues, among which GNAS expression is the highest in the pancreas (Fig. 1a). Additionally, we examined GNAS protein expression level in several hepatoma cell lines. The results showed that GNAS is highly expressed in both HCC cells and the HL-7702 normal liver cells (Fig. 1b), and the protein expression level of GNAS is higher in HepG2 HCC cells. Thus, we used HepG2 cells for the subsequent studies.

Furthermore, we explored whether inflammation would affect GNAS expression in HCC cells. As shown in Fig. 1c and d, LPS or diethylnitrosamine (DEN), a drug commonly used to induce hepatocarcinogenesis in vivo [18], stimulation upregulated GNAS expression in a dose-dependent manner in HepG2 cells. Next, we wondered whether the upregulated expression of GNAS could enhance the inflammatory response in HCC cells. As shown in Fig. 1e, knockdown of GNAS significantly decreased the mRNA expression of IL-6 in HepG2 cells following LPS stimulation, while mRNA levels of TNF-α, IL-1β, IL-8 and IL-10 remained unchanged in LPS-stimulated HepG2 cells. Meanwhile, the protein level of IL-6 in supernatant of HepG2 cells decreased upon knockdown of GNAS (Fig. 1f). Taken together, our results show that LPS stimulation promotes GNAS expression in HCC cells, and GNAS knockdown inhibits LPS-induced IL-6 expression, indicating that GNAS might be involved in the inflammation-related HCC progression.

### LPS stimulation promotes GNAS expression through increasing N^6^-methyladenosine (m6A) methylation of GNAS mRNA in HCC cells

**Fig. 2:**
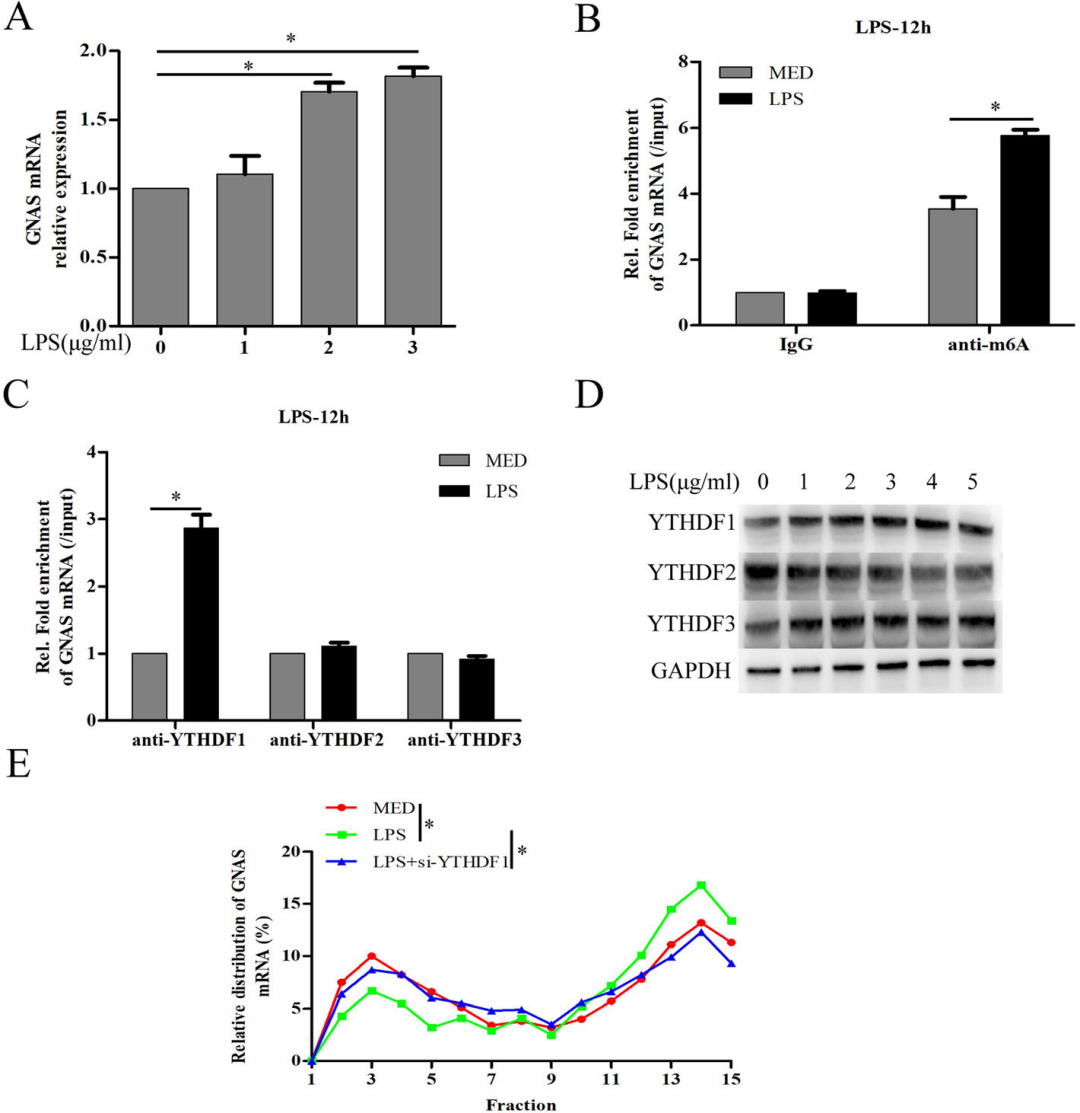
LPS stimulation promotes GNAS expression through increasing N^6^-methyladenosine (m6A) methylation of GNAS mRNA in HCC cells. **a** HepG2 cells were treated with the indicated LPS for 12 h, and then GNAS mRNA expression levels were detected by qRT-PCR. **b** HepG2 cells were treated with LPS (5 μg/ml) or culture medium (MED) for 12 h, and then the m6A modification of GNAS mRNA expression were detected by RIP assay. **c** HepG2 cells were treated with LPS (5 μg/ml) or culture medium (MED) for 12 h, and then the interactions between YTHDF1/2/3 and GNAS mRNA expression were detected by RIP assay. **d** HepG2 cells were treated with LPS or culture medium (MED) for 12 h, and then the protein expression levels of YTHDF1/2/3 were detected by Western blotting. **e** HepG2 cells were transfected with si-NC or si-YTHDF1 for 24 h and then treated with LPS (5 μg/ml) or MED for 12 h. The distribution of endogenous YTHDF1-related GNAS mRNA in the ribosome fractions was detected by polysome profiling RT-PCR experiments. Data are represented as means ± SD (*n* = 3; ^*^represents *P* < 0.05)

To investigate how LPS stimulation promotes the expression of GNAS in HCC cells, the mRNA expression level of GNAS was detected and we found that LPS stimulation significantly upregulated GNAS mRNA expression in HCC cells (Fig. 2a). m6A, the most prevalent internal RNA modification on mammalian messenger RNAs, controls fates and functions of modified transcripts through m6A specific binding proteins [28]. As the best characterized as m6A “readers”, YTH domain containing family 1 (YTHDF1) promotes translation efficiency by binding m6A-modified mRNA [29], whereas YTHDF2 decreases mRNA stability and facilitates mRNA degradation [30]. YTHDF3 facilitates translation and decay of m6A-modified mRNAs through cooperation with YTHDF1 and YTHDF2 [31]. Next, we further investigated whether LPS stimulation-promoted upregulation of GNAS mRNA is related to m6A modification. The results of RNA-binding protein immunoprecipitation (RIP) using anti-m6A antibody showed that LPS stimulation indeed increased the m6A modification on GNAS mRNA (Fig. 2b). Furthermore, LPS stimulation significantly increased YTHDF1, but not YTHDF2 or YTHDF3, binding to GNAS mRNA (Fig. 2c). Moreover, LPS stimulation significantly increased YTHDF1 protein expression in a dose-dependent manner, but slightly decreased YTHDF2 protein expression in HCC cells (Fig. 2d). Next, polysome profiling-RT-PCR experiments were used to examine the distribution of endogenous YTHDF1-related GNAS mRNA in the ribosome fractions to quantify the translated proportion. As shown in Fig. 2e, LPS stimulation promotes, but YTHDF1 knockdown rescues, the transformation from the subpolysome to the polysome fraction. Overall, our results show that LPS stimulation promotes GNAS mRNA translation through increasing m6A methylation of GNAS mRNA in HCC cells.

### GNAS knockdown inhibits LPS induced-IL-6 expression by suppressing STAT3 activation in HCC cells

**Fig. 3:**
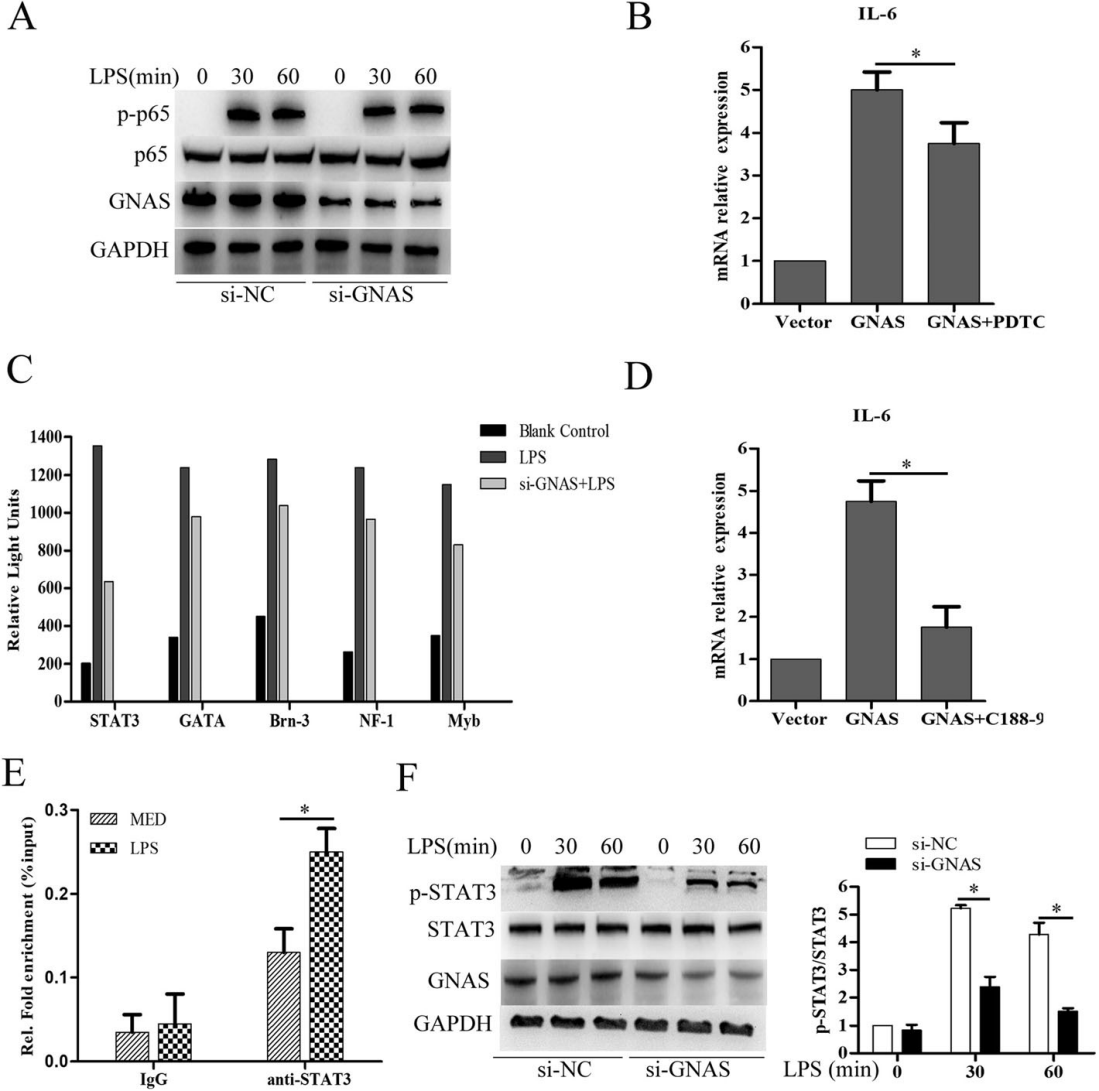
GNAS knockdown inhibits LPS-induced IL-6 expression by suppressing STAT3 activation in HCC cells. **a** HepG2 cells were transfected with si-NC or si-GNAS for 24 h and then treated with LPS (5 μg/ml) or not for the indicated hours. Then, the protein expression levels of p65, phosphorylated p65 (p-p65), and GNAS were detected by Western blotting. **b** HepG2 cells were transfected with pCMV-myc vector or pCMV-myc-GNAS for 24 h, and then treated with the specific inhibitor of NF-kB, PDTC, for 30 min. IL-6 mRNA expression levels were detected by qRT-PCR. **c** HepG2 cells were transfected with si-NC or si-GNAS for 24 h and then treated with LPS or MED for 30 min. Transcription factors activation profiling plate array was performed. **d** HepG2 cells were transfected with pCMV-myc vector or pCMV-myc-GNAS for 24 h, and then treated with a specific inhibitor of STAT3, c188–9, for 30 min. IL-6 mRNA expression levels were detected by qRT-PCR. **e** HepG2 cells were transfected with si-NC or si-GNAS for 24 h and then treated with LPS or MED for 30 min. STAT3 binding on IL-6 promoter was detected by ChIP assay. **f** HepG2 cells were transfected with si-NC or si-GNAS for 24 h and then treated with LPS (5 μg/ml) or not for the indicated hours. Then, the protein expression levels of STAT3, phosphorylated STAT3 (p-STAT3), and GNAS were detected by Western blotting. Data are represented as means ± SD (*n* = 3; ^*^represents *P* < 0.05)

To investigate how GNAS regulates IL-6 expression in HCC cells, phosphorylated NF-κB subunit p65 (p-p65) was detected and we found that GNAS knockdown did not obviously affect LPS stimulation-induced phosphorylation of p65 in HCC cells (Fig. 3a). Consistently, GNAS overexpression significantly promoted IL-6 mRNA expression in HCC cells, whereas treatment with pyrrolidine dithiocarbamate (PDTC), a specific NF-κB inhibitor, only slightly decreased the IL-6 mRNA expression, but did not completely suppress GNAS overexpression-induced IL-6 mRNA expression in HCC cells (Fig. 3b). To further explore the mechanism of GNAS promoting IL-6 expression in HCC cells, the transcription factors activation profiling plate array was performed. As shown in Fig. 3c, GNAS knockdown significantly inhibited LPS-induced activation of STAT3, GATA, Brn-3, NF-1 and Myb, among which STAT3 activation was the most inhibited. Furthermore, treatment with C188–9, a specific STAT3 inhibitor, strongly suppressed GNAS overexpression-induced IL-6 mRNA expression in HCC cells (Fig. 3d). Moreover, chromatin immunoprecipitation (ChIP) assay showed that LPS stimulation significantly promoted STAT3 binding to the IL-6 promoter in HCC cells (Fig. 3e). In addition, GNAS knockdown significantly inhibited LPS-induced phosphorylation of STAT3 (Fig. 3f). Overall, GNAS knockdown inhibits LPS induced-IL-6 expression by suppressing STAT3 activation in HCC cells.

### GNAS promotes LPS-induced HCC cell growth and invasion

**Fig. 4:**
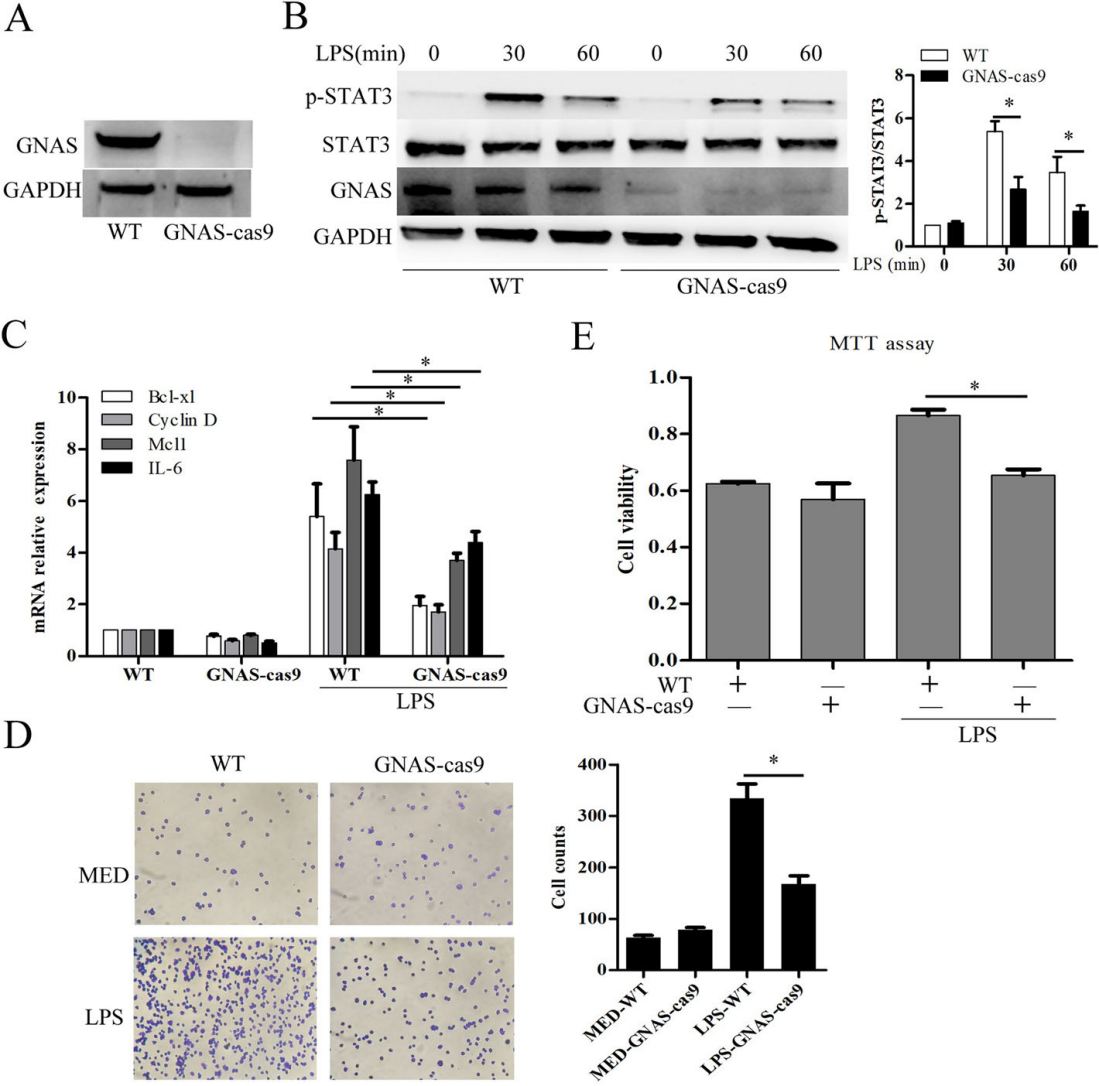
GNAS promotes LPS-induced HCC cell growth and invasion. **a** Protein expression levels of GNAS in wild type HepG2 cells or GNAS knockout-HepG2 cells were detected by Western blotting. **b** HepG2 cells or GNAS knockout-HepG2 cells were treated with LPS (5 μg/ml) or not for the indicated hours. Then, the protein expression levels of STAT3, p-STAT3, and GNAS were detected by Western blotting. **c**, **d** and **e** HepG2 cells or GNAS knockout-HepG2 cells were treated with LPS (5 μg/ml) or not for 12 h. Then, the mRNA expression levels of the indicated genes were detected by qRT-PCR (**c**). Cell invasive ability was examined by transwell invasion assays (**d**). Cell proliferation was examined by MTT assays (**e**). Data are represented as means ± SD (*n* = 3; ^*^represents *P* < 0.05)

We next evaluated the impact of GNAS on inflammation-induced HCC progression. Firstly, we generated a GNAS knockout HepG2 cell line by the CRISPR/Cas9 method (Fig. 4a). Consistent with the results of Figs. 1e and 3f, GNAS knockout significantly suppressed LPS-induced STAT3 phosphorylation (Fig. 4b), and inhibited LPS-induced STAT3 downstream expression of genes such as Bcl-xl, cyclin D, Mcl1 and IL-6 in HCC cells (Fig. 4c). Subsequently, we examined the impact of GNAS on LPS-induced HCC cell growth and invasion. In Matrigel invasion assays, GNAS knockout significantly impaired LPS-induced HCC cell invasion (Fig. 4d). Furthermore, GNAS knockout significantly suppressed LPS-induced HCC cell proliferation (Fig. 4e). Overall, these results indicate that GNAS promotes LPS-induced HCC cell growth and invasion.

### GNAS promotes LPS-induced HCC cell growth and invasion by interacting with STAT3

**Fig. 5:**
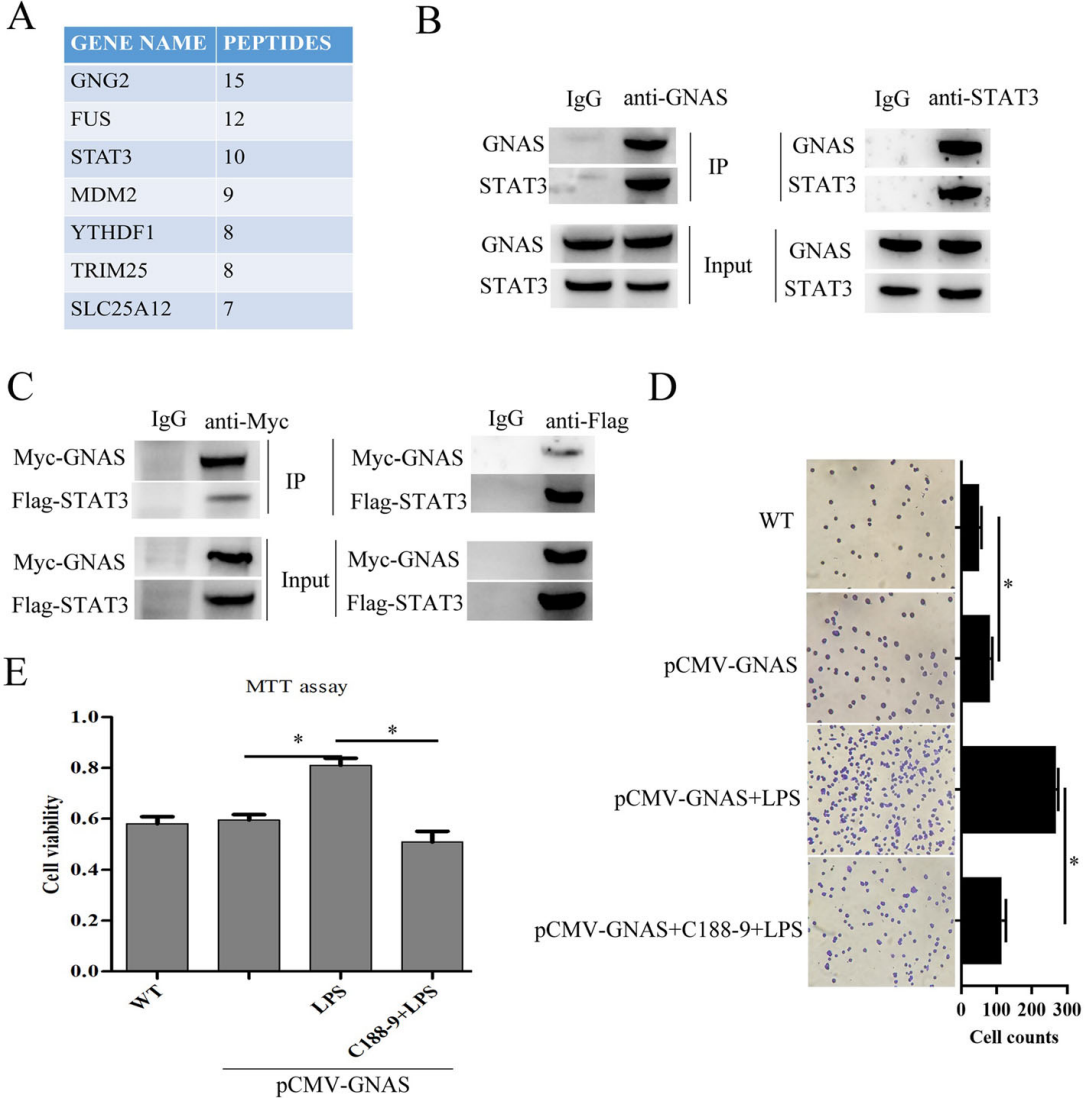
GNAS promotes LPS-induced HCC cell growth and invasion by interacting with STAT3. **a** Table listing the GNAS-interacting proteins that coimmunoprecipitated with anti-GNAS antibody from HepG2 cells identified by mass spectrometry. **b** The interactions of endogenous GNAS and endogenous STAT3 in HepG2 cells were detected by CO-IP. **c** HepG2 cells were transfected with pCMV-myc-GNAS and pCMV-flag-STAT3 for 24 h, and then the interactions of exogenous GNAS and exogenous STAT3 were detected by CO-IP. **d** and **e** HepG2 cells were transfected with pCMV-myc-GNAS for 24 h, treated with a specific inhibitor of STAT3, c188–9, for 30 min, and then stimulated with LPS (5 μg/ml) for 12 h. Cell invasive ability was examined by transwell invasion assays (**d**). Cell proliferation was examined by MTT assays (**e**). Data are represented as means ± SD (*n* = 3; ^*^represents *P* < 0.05)

To further explore the molecular mechanism of GNAS promoting LPS-induced HCC cell growth and invasion, Co-IP accompanied by mass spectrometry was performed to identify the GNAS-interacting proteins in HCC cells. Among the potential interacting proteins (Fig. 5a), we focused on STAT3 in the subsequent studies, due to the fact that STAT3 has been proved to play critical roles in driving the proliferation, invasiveness, and metastasis of cancer cells [10, 32]. Subsequently, the interaction between endogenous/exogenous GNAS and STAT3 was confirmed by co-immunoprecipitation assays (Fig. 5b and c). To verify whether GNAS promoting LPS-induced HCC cell growth and invasion is related to its interacting with STAT3, HCC cells were transfected with pCMV-GNAS plasmid and then treated with LPS and/or C188–9. As shown in Fig. 5d, inhibiting STAT3 with C188–9 significantly suppressed GNAS overexpression, promoting LPS-induced HCC cell invasion. In addition, STAT3 inhibition also significantly suppressed GNAS overexpression, promoting LPS-induced HCC cell proliferation (Fig. 5e). Taken together, these results demonstrate that GNAS promotes LPS-induced HCC cell growth and invasion by interacting with STAT3.

### GNAS promotes LPS-induced STAT3 activation in HCC cells through inhibiting long non-coding RNA TPTEP1 interacting with STAT3

**Fig. 6:**
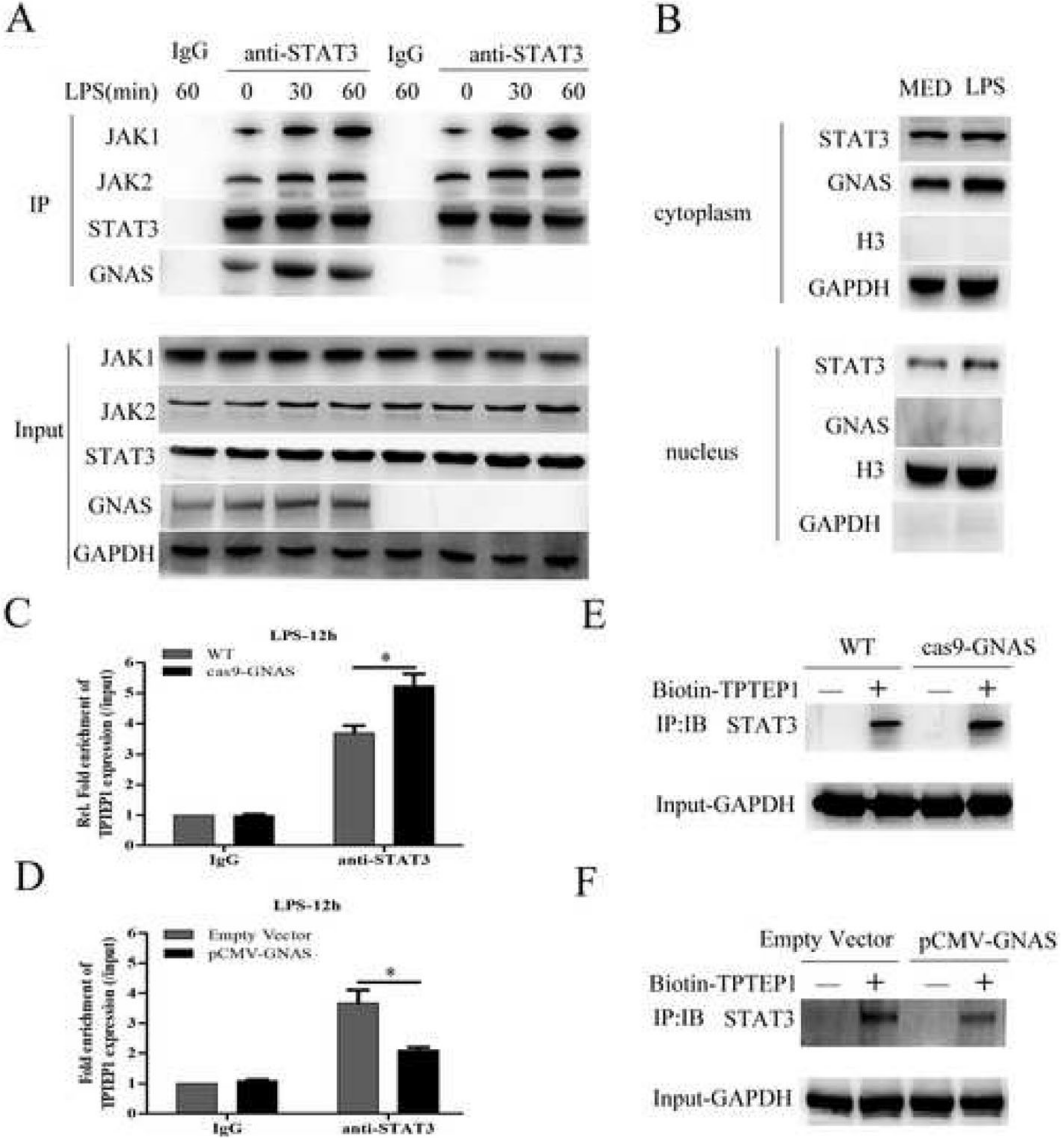
GNAS promotes LPS-induced STAT3 activation in HCC cells through inhibiting long non-coding RNA TPTEP1 interacting with STAT3. **a** HepG2 cells or GNAS knockout-HepG2 cells were treated with LPS (5 μg/ml) or not for the indicated hours. Then, the interactions of JAK1/2, STAT3 and GNAS were detected by CO-IP. **b** HepG2 cells were treated with LPS (5 μg/ml) or MED for 12 h, and then the protein expression levels of STAT3 and GNAS in the cytoplasmic and nuclear fractions were detected by Western blotting (GAPDH as the cytoplasmic marker, and histone H3 as the nuclear marker). **c** HepG2 cells or GNAS knockout-HepG2 cells were treated with LPS (5 μg/ml) or not for 12 h. The interaction between STAT3 and TPTEP1 was detected by RIP. **d** HepG2 cells were transfected with pCMV-myc vector or pCMV-myc-GNAS for 24 h, and then treated with LPS (5 μg/ml) or not for 12 h. The interaction between STAT3 and TPTEP1 was detected by RIP. **e** The interaction between biotin-labeled TPTEP1 and STAT3 in HepG2 cells or GNAS knockout HepG2 cells was detected by RNA pull-down. **f** The interaction between biotin-labeled TPTEP1 and STAT3 in HepG2 cells or GNAS overexpressed-HepG2 cells was detected by RNA pull-down. Data are represented as means ± SD (*n* = 3; ^*^represents *P* < 0.05)

To further investigate the underlying mechanism of GNAS promoting LPS-induced STAT3 activation in HCC cells, we examined the effects of GNAS on JAK-STAT3 signaling. As shown in Fig. 6a, during the process of LPS stimulation, the interactions of STAT3 and JAK1, JAK2 or GNAS were significantly increased, and GNAS knockout did not prominently affect the interactions between STAT3 and JAK1 or JAK2, indicating that GNAS promoting LPS-induced STAT3 activation is not related to the upstream factors of STAT3. Next, to explore where GNAS interacts with STAT3 in HCC cells, we isolated the cytosolic and nuclear fractions from LPS-stimulated HCC cells. Immunoblot showed that GNAS was mainly distributed in the cytoplasm, which indicates that GNAS interacts with STAT3 in the cytoplasm, but not in the nucleus (Fig. 6b). Our recent study reported that long non-coding RNA TPTEP1 inhibits hepatocellular carcinoma progression by interacting and suppressing STAT3 phosphorylation [25]. Furthermore, we investigated whether GNAS would affect the interaction between TPTEP1 and STAT3 in HCC cells. As shown in Fig. 6c and d, GNAS knockout significantly promoted the interaction of TPTEP1 and STAT3 in LPS-stimulated HCC cells, and GNAS overexpression evidently inhibited it, as detected by RIP. Consistently, RNA pull-down assays also confirmed that GNAS knockout promoted, and GNAS overexpression inhibited, the interaction of biotin-tagged TPTEP1 and STAT3 in HCC cells (Fig. 6e and f). Overall, GNAS promotes LPS-induced STAT3 activation in HCC cells through inhibiting long non-coding RNA TPTEP1 interacting with STAT3.

### GNAS expression contributes to HCC development in mice and is related to poor survival

**Fig. 7:**
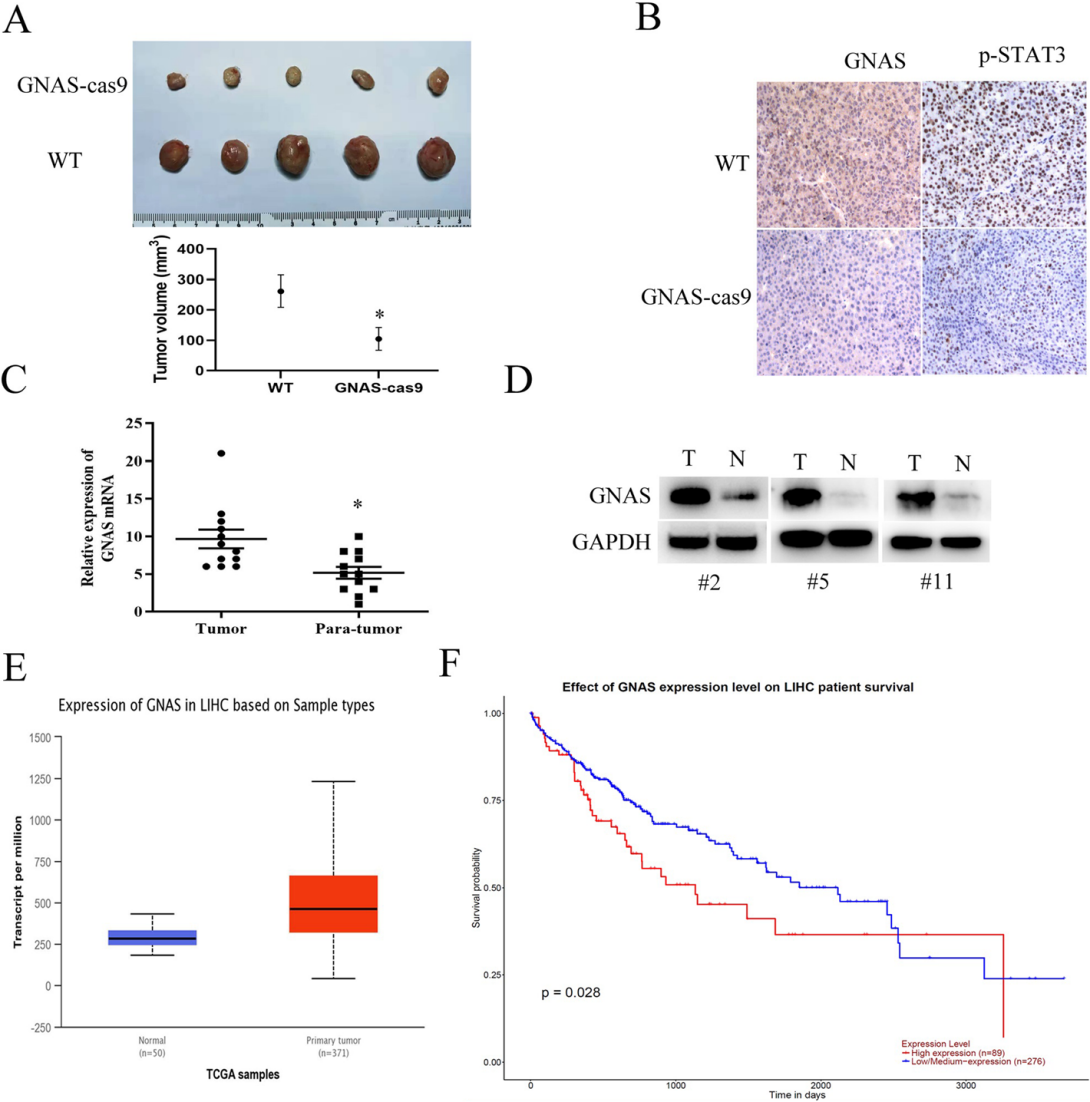
GNAS expression contributes to HCC development in mice and is related to poor survival. **a** GNAS knockout significantly inhibits tumor growth in vivo. Representative images of xenograft tumors from the nude mice. **b** IHC analysis of GNAS and p-STAT3 in tumor tissues (× 200 magnification). mRNA (**c**) and protein (**d**) expression analysis of GNAS in clinical tumor and para-tumor normal tissue samples. **e** GNAS mRNA expression analysis using TCGA database (*p* < 1E-12). **f** Survival analysis of high or low expression of GNAS on liver hepatocellular carcinoma patient from TCGA database (*p* = 0.028)

To investigate the tumorigenesis effect of GNAS in vivo, we subcutaneously injected wild type (WT) or GNAS-knockout (GNAS-cas9) HepG2 cells into nude mice and found that GNAS knockout caused less tumor formation and significantly decreased tumor size compared with the WT group (Fig. 7a). Additionally, immunochemical analysis showed that p-STAT3 expression was evidently decreased in the tumor tissues of the GNAS-cas9 group, compared to that in the WT group (Fig. 7b). Furthermore, we detected the GNAS mRNA or protein expression levels in clinical HCC tissue samples. As shown in Fig. 7c and d, GNAS mRNA and protein expression levels were higher in tumor tissues, compared with those in the corresponding para-tumor normal tissues. Moreover, we analyzed the TCGA database and found that GNAS is relatively highly expressed in liver hepatocellular carcinoma, compared to normal tissue (Fig. 7e), and highly expressed GNAS is related to poor survival (Fig. 7f). Overall, these results indicate that GNAS is frequently upregulated in HCC tissues and promotes tumor masses.

## Discussion

Hepatocellular carcinoma is a serious disease contributing to global death annually [2]. Due to the limited therapeutic efficacy in clinical practice, clarifying the complicated molecular mechanism involved in HCC is urgent for developing new therapeutic methods. In this study, we focused on the IL-6/STAT3 signaling required for HCC development and explored the role of GNAS in inflammation-related HCC. GNAS participates in LPS-induced HCC proliferation and invasion by promoting IL-6/STAT3 signaling. GNAS knockdown inhibits STAT3 phosphorylation by strengthening the inhibitory effect of TPTEP1 on STAT3. Our previous study had demonstrated that long non-coding RNA TPTEP1 interacts with the DNA binding domain (DBD) of STAT3 protein to inhibit STAT3 activation in HCC cells [25]. For further research, we aim to clarify the molecular structural basis of GNAS inhibiting TPTEP1 binding to STAT3. Moreover, the functional phosphorylation site of STAT3 Y705 is located in the tail domain, which is far from the DBD domain, and GNAS promotes STAT3 Y705 phosphorylation partly through TPTEP1, which indicates that GNAS might affect Y705 phosphorylation through interacting with the DBD domain. Whether other sites of DBD region modifications or spatial structure approximation affect Y705 phosphorylation needs to be investigated. From Jean’s work, IL-6 and interferon pathways were activated in GNAS-mutated tumor tissues [23], suggesting that GNAS enzymatic activity is necessary for IL-6/STAT3 activation. However, our study demonstrated the sequestration function of GNAS during STAT3 phosphorylation, which seems contradictory to this report. Combined with our results, it may suggest that enzymatic activity of GNAS is required for STAT3 activation, but GNAS-activating mutation strengthens the association with STAT3, which relieves the inhibitory effect of TPTEP1 on STAT3. In view of the fact that the DBD domain of STAT3 is required for DNA binding in the nucleus and the DBD domain may be necessary for GNAS-mediated STAT3 phosphorylation regulation in the cytoplasm, we wonder whether the DBD domain is another regulatory center when STAT3 locates in the cytoplasm and shifts to the DNA binding function when STAT3 translocates into the nucleus, which indicates that the same domain may have vastly different function owing to the different location.

## Conclusion

Our study explores the regulatory role of GNAS during STAT3 phosphorylation in HCC cells and demonstrates that GNAS promotes STAT3 Y705 phosphorylation by inhibiting TPTEP1 binding to STAT3, which mediates inflammation-induced hepatocellular carcinoma cell lines’ proliferation and invasion. Our findings for the first time suggest a tumor-promoting role of GNAS in inflammation-related HCC progression and provide a novel potential target for HCC therapy.

## Supplementary information

**Additional file 1.** Raw images from western blots.

## Data Availability

The datasets used and/or analyzed during the current study are available from the corresponding author on reasonable request.

## Abbreviations

ChIP: Chromatin immunoprecipitation
Co-IP: Co-immunoprecipitation
DEN: Diethylnitrosamine
ELISA: Enzyme-linked immunosorbent assay
GNAS: G-protein alpha-subunit
HCC: Hepatocellular carcinoma
IL-6: interleukin-6
LPS: Lipopolysaccharides
m6A: N6-methyladenosine
PDTC: Pyrrolidine dithiocarbamate
qRT-PCR: Quantitative real-time PCR
RIP: RNA-binding protein immunoprecipitation
STAT3: Signal transducer and activator of transcription 3
TGF-β: Transforming growth factor-beta
TNF-α: Tumor necrosis factor alpha
YTHDF1: YTH domain containing family 1
