# Supplementary material for: GNAS promotes inflammation-related hepatocellular carcinoma progression by promoting STAT3 activation
Source: Cell Mol Biol Lett. 2020 Feb 24;25:8. doi: 10.1186/s11658-020-00204-1 (PMC7038622; doi:10.1186/s11658-020-00204-1)
Supplement: Supplementary file 1 — Additional file 1. Raw images from western blots. [file 11658_2020_204_MOESM1_ESM.doc]

Fig.1A

GNAS GAPDH


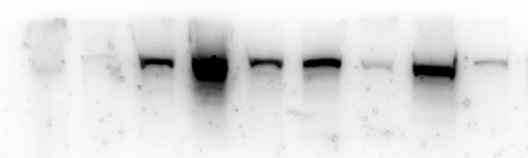

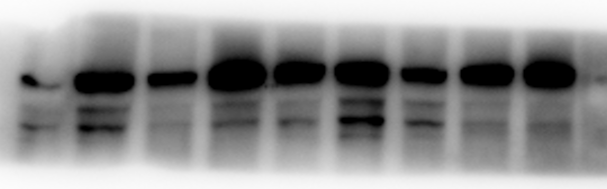


Fig.1B

GNAS GAPDH


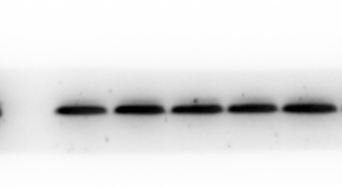


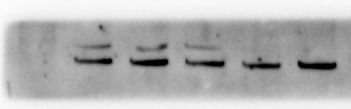


Fig.1C

GNAS GAPDH


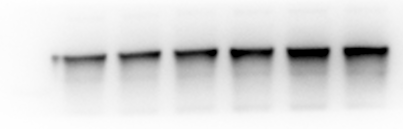

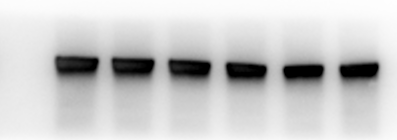


Fig.1D

GNAS GAPDH


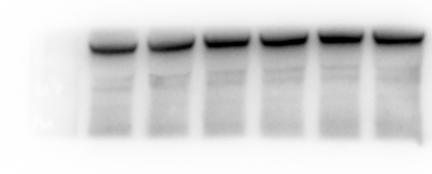

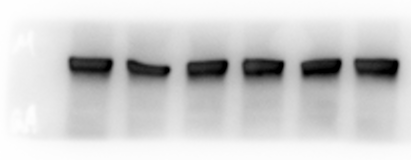


Fig.2D

YTHDF 1 YTHDF 2


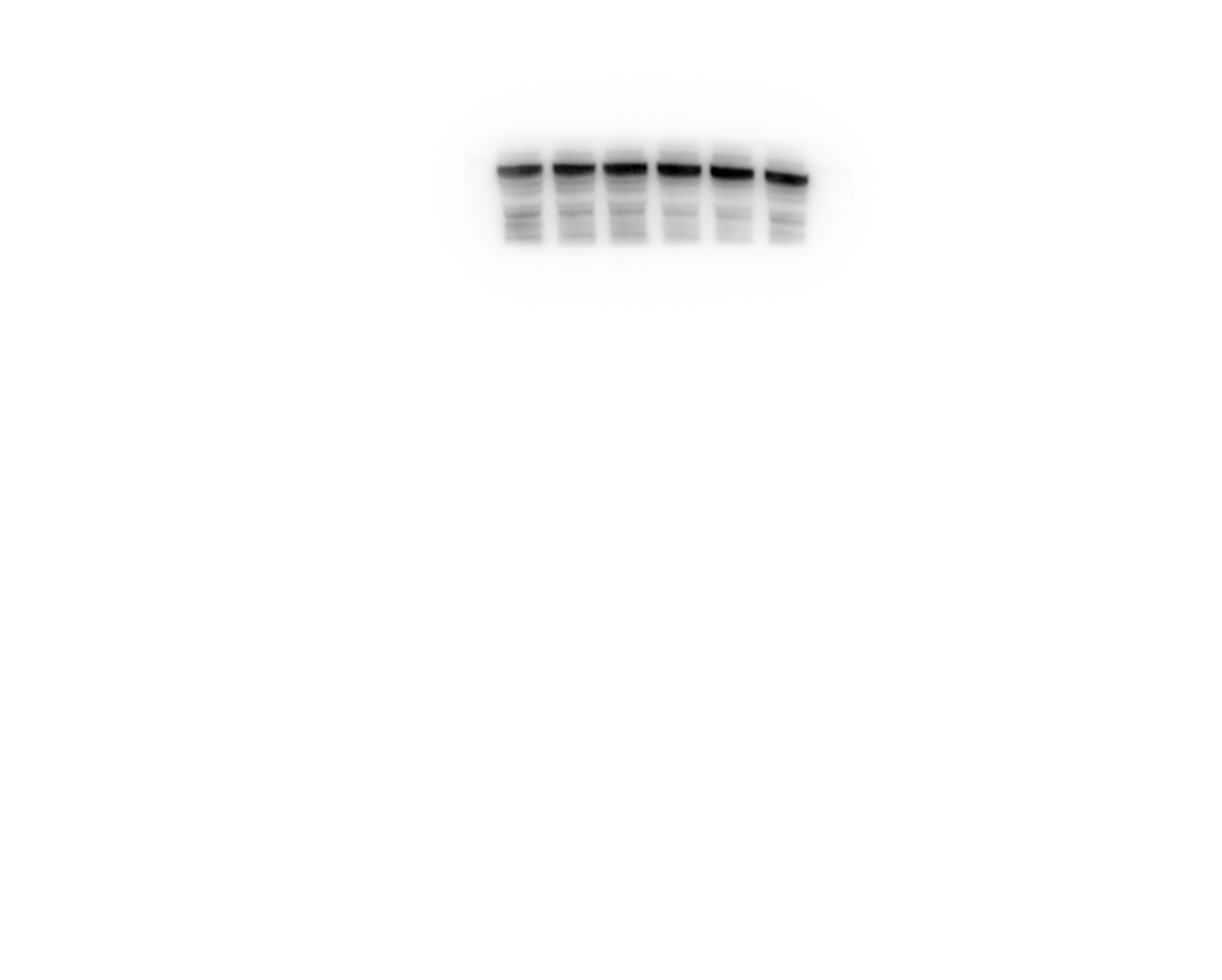

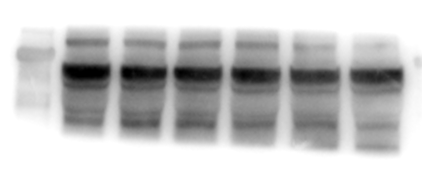


YTHDF 3 GAPDH


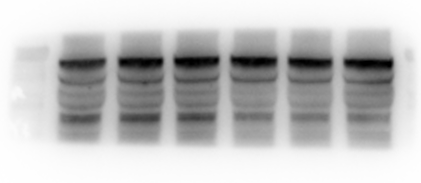


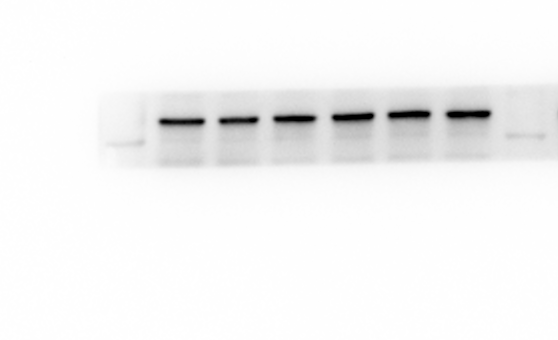


Fig.3A

P-p65 p65


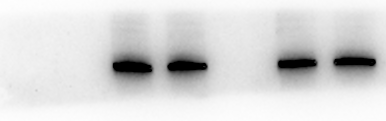

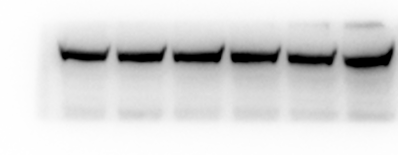


GNAS GAPDH


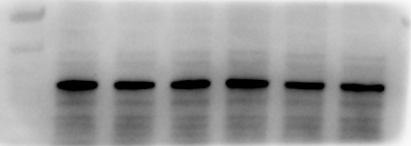


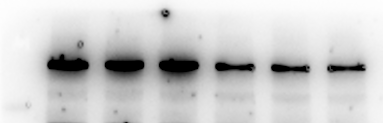


Fig.3F

P-stat3 stat3


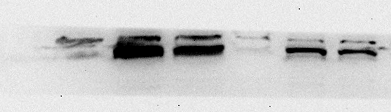


GNAS GAPDH


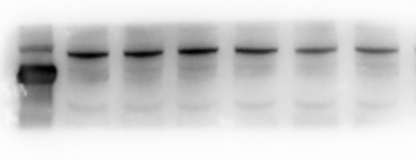

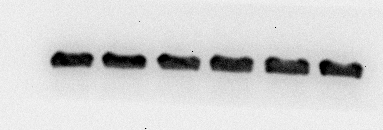


Fig.4A

GNAS GAPDH


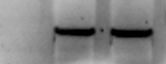

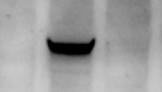


Fig.4B


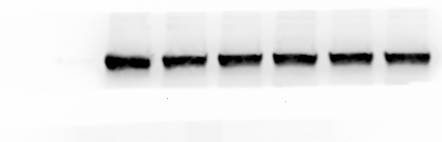
P-stat3 stat3


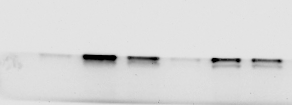


GNAS GAPDH


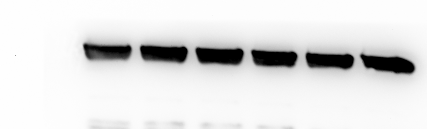


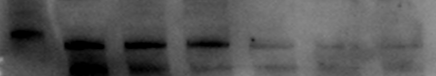


Fig.5B

GNAS-input(IgG anti-GNAS) stat3-input(IgG anti-GNAS)


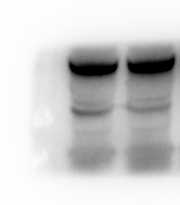


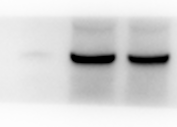


GNAS-ip(IgG anti-GNAS) stat3-ip(IgG anti-GNAS)


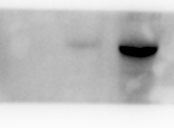


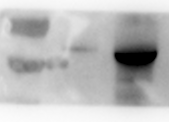


GNAS-input(IgG anti-stat3) stat3-input(IgG anti-stat3)

GNAS-ip(IgG anti-stat3) stat3-ip(IgG anti-stat3)


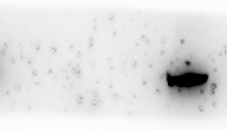


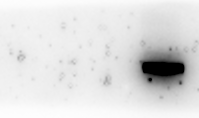


Fig.5C

Myc-GNAS-input(IgG anti-Myc) Flag-stat3-input(IgG anti-Myc)


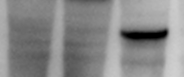


Myc-GNAS-ip(IgG anti-Myc) Flag-stat3-ip(IgG anti-Myc)


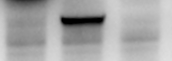


Myc-GNAS-input(IgG anti-flag) Flag-stat3-input(IgG anti-flag)


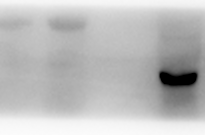

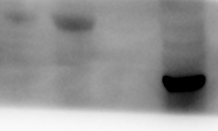


Myc-GNAS-ip(IgG anti-Flag) Flag-stat3-ip(IgG anti-Flag)


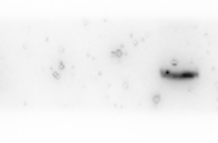


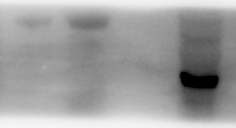


Fig.6A

Input-GAPDH input-GNAS


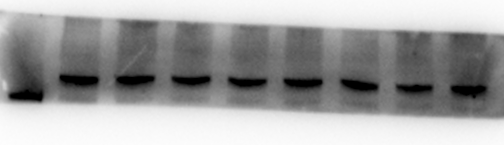

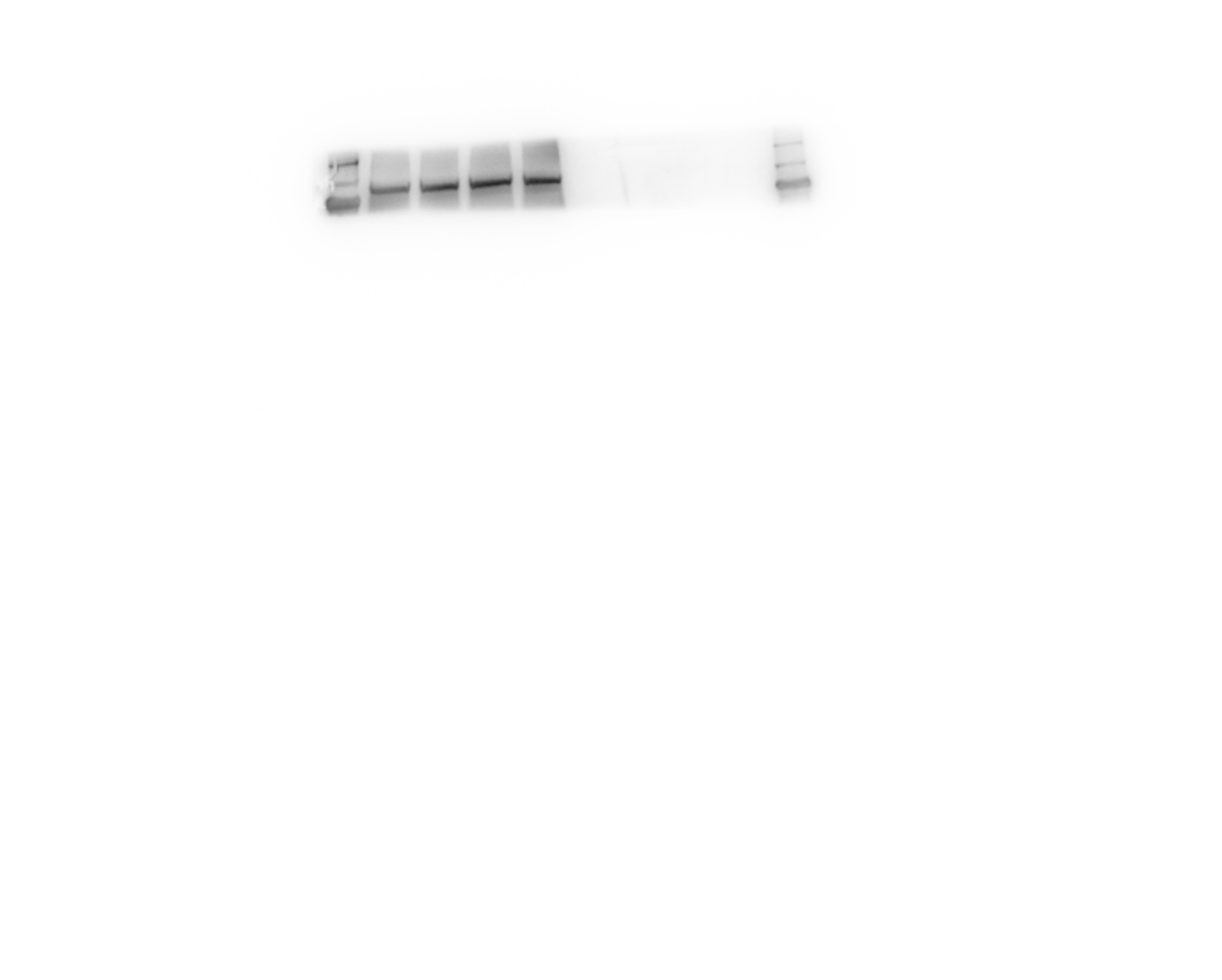


Input-JAK1 input-JAK2


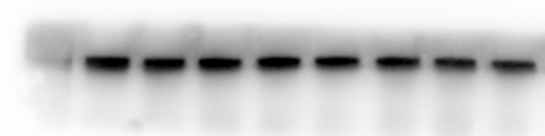

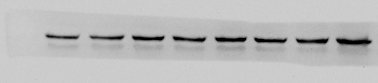


Input-stat3

Ip-GNAS Ip-stat3


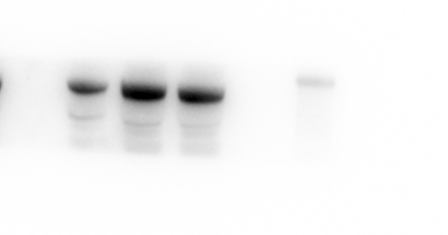


Ip-JAK1 Ip-JAK2

Fig.6B

Cytoplasm-stat3(med-LPS) Cytoplasm-GNAS(med-LPS) Cytoplasm-GAPDH(med-LPS)


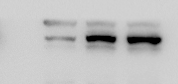

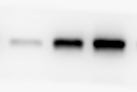

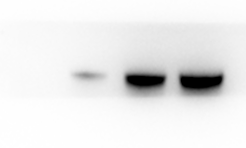


Cytoplasm-H3(med-LPS) nucleus-stat3(med-LPS) nucleus-gnas(med-LPS)


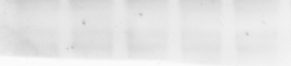

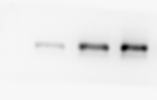

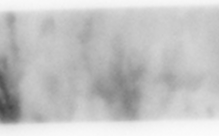


nucleus-gapdh(med-LPS) nucleus-H3(med-LPS)


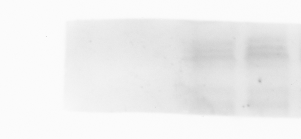


Fig.6E


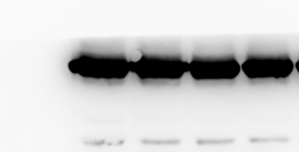
Input-gapdh STAT3


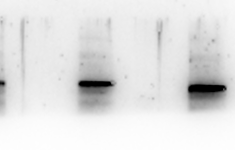


Fig.6F

Input-gapdh STAT3


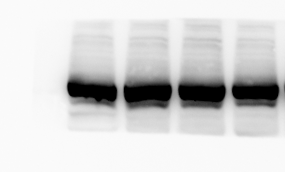

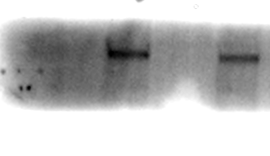


Fig.7D

GNAS(#2) GAPDH(#2)

GNAS(#5) GAPDH(#5)

GNAS(#11) GAPDH(#11)
